# Propranolol Administration Modulates Neural Activity in the Hippocampal Hilus During Fear Retrieval

**DOI:** 10.3389/fnbeh.2022.919831

**Published:** 2022-07-07

**Authors:** Sofia Leal Santos, Briana K. Chen, Guilherme R. Pereira, Vananh Pham, Christine A. Denny

**Affiliations:** ^1^Department of Psychiatry, Columbia University Irving Medical Center, New York, NY, United States; ^2^Division of Systems Neuroscience, Research Foundation for Mental Hygiene, Inc. (RFMH)/New York State Psychiatric Institute (NYSPI), New York, NY, United States; ^3^Life and Health Sciences Research Institute (ICVS), School of Medicine, University of Minho, Braga, Portugal; ^4^Instituto de Investigação em Ciências da Vida e da Saúde (ICVS)/3Bs - PT Government Associate Laboratory, Guimarães, Portugal; ^5^Neurobiology and Behavior (NB&B) Graduate Program, Columbia University, New York, NY, United States

**Keywords:** hippocampus, hilus, Arc, Fos, somatostatin, parvalbumin

## Abstract

Altered fear learning is a strong behavioral component of anxiety disorders such as post-traumatic stress disorder (PTSD). Recent efforts have attempted to combine exposure therapies with drugs that target fear memory retrieval and memory reconsolidation, in order to improve treatment efficacy. The noradrenergic (NA) signaling system is of particular interest, due to its role in regulating the stress response and its involvement in fear and learning processes. Importantly, propranolol (P), a non-selective β-adrenergic antagonist, has shown the potential in decreasing exaggerated fear in both humans and animal models. In a previous study, we utilized an activity-dependent tagging murine model to determine the neural mechanisms by which propranolol attenuates learned fear. We found that propranolol acutely decreased memory trace reactivation specifically in the dorsal dentate gyrus (dDG), but not in CA3 or CA1. Here, we extended our previous study by investigating whether propranolol additionally altered activity in the hilus, a polymorphic layer that consists of neurons, mossy cells, and GABAergic interneurons. We found that propranolol acutely reduced overall hilar activity in both the dorsal and ventral hilus. Moreover, we report that propranolol significantly altered the activity of parvalbumin (PV)^+^ cells in the ventral (vDG), but not dorsal DG (dDG). Together, these results suggest that a β-adrenergic blockade may affect the activity of excitatory and inhibitory cell types in the hilar layer of the DG, and that these alterations may contribute to manipulating fear memory traces.

## Introduction

Post-traumatic stress disorder (PTSD) and other anxiety disorders such as specific phobias, often include abnormally strong fear responses that are resistant to therapeutic approaches with exposure therapies (Rauch et al., 2012; Botella et al., 2017). Numerous efforts have been made to couple exposure therapies for PTSD with pharmacological manipulations to successfully manage exaggerated fear responses (Taylor et al., 2008; Belkin and Schwartz, 2015; Brunet et al., 2018). While research is still ongoing, some results suggest that this combinatorial approach may result in effective, positive clinical outcomes for managing fear and anxiety disorders.

One of the targets of this approach is the noradrenergic system, which is known to be involved in vigilance, attention, threat response, and in learning and memory (Berridge and Waterhouse, 2003; Berridge, 2005; Avery and Krichmar, 2017). The main source of noradrenergic innervation in the brain is the locus coeruleus (LC), a brain nucleus in the pons of the brain stem, and part of the reticular ascending system (Berridge and Waterhouse, 2003; Schwarz and Luo, 2015; Aston-Jones and Waterhouse, 2016). Drugs that manipulate adrenergic signaling have been used to manipulate either the reactivation, the reconsolidation, and/or the extinction processes involved in the weakening of a fearful memory (Taylor et al., 2008; Lonergan et al., 2013; McGuire et al., 2014; Belkin and Schwartz, 2015). The non-selective β-adrenergic antagonist propranolol (P), in particular, has been used for several decades in anxiety disorders including performance anxiety (Tyrer, 1988; Liu et al., 1991; Soeter and Kindt, 2015) and generalized anxiety disorder (GAD) (Fagerström et al., 1985; Tyrer, 1988; Grillon et al., 2004). Propranolol has also been used to aid in the treatment of phobias and in exposure therapies for PTSD (Grillon et al., 2004; Soeter and Kindt, 2015; Kroes et al., 2016).

To better understand the mechanism of action of propranolol, it is critical to test the drug’s effects in different behavioral situations, with varied conditioned stimuli and administration timelines, while analyzing the underlying brain activity. Of particular interest are several regions that are involved in the learning of fearful associative memories, and that also receive abundant noradrenergic projections from the LC. These include the hippocampal formation (HPC), the medial prefrontal cortical areas prelimbic (PL) and infralimbic areas (ILA), and several amygdalar nuclei, the most studied of which are the basolateral (BLA) and the lateral (LA) amygdalar nuclei, all of which have been shown to be affected by propranolol administration in humans (Hurlemann et al., 2010; Schwabe et al., 2012; Kroes et al., 2016) and in animal models (Ji et al., 2003; Chen and Sara, 2007; Do Monte et al., 2008; Qi et al., 2008; Ehrlich et al., 2009; Rodriguez-Romaguera et al., 2009; Vetere et al., 2013; Fitzgerald et al., 2015; Hagena et al., 2016; Giustino et al., 2017).

Engrams or memory traces were conceptualized by Richard Semon in the early twentieth century (Semon, 1921) and refer to the change in biological matter that occurs with learning, that is maintained, and whose reactivation leads to retrieval of the memory. In the past decade, numerous studies have utilized the expression of immediate early genes (IEGs), which undergo an increase in transcription when a neuron has increased activity, as a proxy for engrams (Tonegawa et al., 2015). By utilizing IEG promoters in inducible, transgenic systems, it became possible to tag a population of neurons active during an individual memory. These recent genetic techniques, paired with optogenetics, have allowed scientists to identify and manipulate engrams (Liu et al., 2012; Denny et al., 2014; Redondo et al., 2014; Ramirez et al., 2015; Roy et al., 2016; Perusini et al., 2017; Khalaf et al., 2018; Chen et al., 2019; Lacagnina et al., 2019), and even may be modified to improve mood and cognition (Reijmers et al., 2007; Denny et al., 2017).

One of these IEGs *Arc/Arg3.1* has been widely implicated in synaptic plasticity (Link et al., 1995; Lyford et al., 1995; Pastuzyn et al., 2018). We previously created the ArcCreER^T2^ x enhanced yellow fluorescent protein (eYFP) mice, which allow for the permanent labeling of activated Arc^+^ neurons (Denny et al., 2014). Using this transgenic murine line, we have shown that the HPC subregions dentate gyrus (DG) and cornus ammonis 3 (CA3) encode memory traces of contextual fear memories (Denny et al., 2014). This work showed that, for both the DG and CA3: (1) the number of tagged cells was greater in mice following CFC compared to mice that underwent exposure to the context with no shock; (2) that the percentage of reactivated cells was higher when mice were re-exposed to the CFC context, compared to mice exposed to a different context; and (3) that the neuronal ensembles active upon encoding of contextual fear conditioning (CFC) memories are necessary for memory retrieval (Denny et al., 2014; Cazzulino et al., 2016; Mastrodonato et al., 2018; Lacagnina et al., 2019). In a more recent study (Leal Santos et al., 2021), we utilized the ArcCreER^T2^ mouse model to study the effects of propranolol in the DG, CA3, and CA1, in prefrontal cortical areas, and in several amygdalar nuclei. We found that propranolol administered immediately before fear retrieval decreased fear expression, which was paralleled by a decreased reactivation of the fearful memory trace specifically in the dorsal DG (dDG).

Here, we sought to further our prior study and to investigate how propranolol administration impacts activity in the hilus. The hilus is the layer of the DG that receives the highest density of afferents of cholinergic, serotoninergic, and, importantly, noradrenergic fibers (Pickel et al., 1974; Swanson and Hartman, 1975), suggesting an important role in mediating how these stimuli affect the activity of the granule cells. Using the ArcCreER^T2^ x eYFP mice to tag fear memory traces, we report that propranolol administration significantly reduced c-Fos expression in both the dorsal and ventral hilus. Propranolol did not alter the expression of either somatostatin (SST) or parvalbumin (PV), markers of inhibitory interneurons. Propranolol administration increased the activity of PV-expressing cells in the ventral hilus. Together, these results suggest that a β-adrenergic blockade may affect the activity of excitatory and inhibitory cell types in the hilar layer of the DG and that these alterations may contribute to reducing fear behavior.

## Materials and Methods

### Mice

ArcCreER^T2^(+) (Denny et al., 2014) × R26R-STOP-floxed-enhanced yellow fluorescent protein (eYFP) (Srinivas et al., 2001) homozygous female mice were bred, reared, and tested as previously described (Leal Santos et al., 2021). Food and water were provided *ad libitum*. All experiments were approved by the Institutional Animal Care and Use Committee (IACUC) at the New York State Psychiatric Institute (NYSPI).

### Drugs

#### 4-Hydroxytamoxifen

4-Hydroxytamoxifen (4-OHT) (Sigma, St. Louis, MO, H7904) was used to induce recombination, as previously described (Cazzulino et al., 2016). 4-OHT was dissolved by sonication in 10% EtOH/90% corn oil at a concentration of 10 mg/ml. One injection of 200 μl (2 mg) was administered intraperitoneally (i.p.) into each adult mouse.

#### Propranolol

(+)-Propranolol hydrochloride was dissolved in 0.9% NaCl at a concentration of 1 mg/ml. A single injection of saline (Sal) (0.9% NaCl) or propranolol hydrochloride (P) [(+)-Propranolol hydrochloride, Sigma Aldrich, #PHR1308] (10 mg/kg) was administered i.p. once immediately before re-exposure to the CFC context. Dosing was based on previous studies, indicating that 10 mg/kg of propranolol was effective at decreasing fear expression acutely or in subsequent re-exposures in mice (Do Monte et al., 2008; Ehrlich et al., 2009; Do-Monte et al., 2010; Muravieva and Alberini, 2010; Fitzgerald et al., 2015; Giustino et al., 2017).

### Behavioral Tests

#### Contextual Fear Conditioning

A 4-shock CFC paradigm and context re-exposure (RE) was administered as previously described (Leal Santos et al., 2021).

#### Tissue Processing

Mice were deeply anesthetized, and perfusions with 4% paraformaldehyde (PFA) and brain processing were performed as previously described in Denny et al. (2014), Cazzulino et al. (2016), Pavlova et al. (2018), and Leal Santos et al. (2021).

#### Immunohistochemistry

An iDISCO-based immunohistochemistry protocol was performed as previously described (Pavlova et al., 2018; Leal Santos et al., 2021). All antibodies and corresponding information are listed in Supplementary Table 1.

#### Confocal Microscopy

All samples were imaged on a confocal scanning microscope (Leica TCS SP8, Leica Microsystems Inc., Wetzlar, Germany) with 2 PMT detectors, as previously described (Leal Santos et al., 2021). Sections were imaged with a dry Leica 20 × objective (NA 0.70, working distance 0.5 mm), with a pixel size of 1.08 × 1.08 μm^2^, a z step of 3 μm, and z-stack of 27 μm. Fields of view were stitched together to form tiled images by using an automated stage and the tiling function and algorithm of the LAS X software.

#### Cell Quantification

An investigator blind to treatment counted eYFP^+^, c-Fos^+^, SST, or PV immunoreactive cells bilaterally in the hilus (also called polymorphous layer of the DG) of the hippocampus. Cells were counted bilaterally using Fiji (Schindelin et al., 2012). A minimum of 3 hemi-sections were analyzed per mouse per dorsal or ventral hilus. The limits of the hilus were defined using the coronal Allen Brain Atlas as a reference. Cell counts were normalized to the volume of hilus for each mouse. Normalized levels per mm^3^ and the reactivation levels (percentage of co-labeled cells) are presented throughout.

#### Statistical Analysis

All data were analyzed using Prism 9.0.0. Alpha was set to 0.05 for all analyses. Normality was tested using the D’Agostino–Pearson test. As normality was present, the effect of drug on average levels of immunolabeled cells was analyzed using *t*-tests. Pearson correlations between levels of activity in the hilus and freezing levels were calculated.

## Results

### Propranolol Administration Decreases Dorsal Hilar Activity

In our previous study, we reported differences in dDG memory trace reactivation following propranolol administration (Leal Santos et al., 2021). Importantly, we quantified cells active during fear encoding and fear memory retrieval in the granule cell layer (GCL) of the dDG, but we did not analyze other layers of the DG, such as the polymorphous layer (PML), which has also been referred to as CA4 or the hilus (De Nó, 1934). Although most excitatory cells in the DG are localized in the GCL, the hilus harbors important excitatory cells (Scharfman and Myers, 2012; Scharfman, 2016) and several populations of inhibitory interneurons (Amaral et al., 2007; Scharfman, 2016). Most notably, the hilus receives the highest density of afferents of cholinergic, serotoninergic, and, importantly, noradrenergic fibers (Pickel et al., 1974; Swanson and Hartman, 1975) and is the layer with the highest expression of β-adrenergic receptors (Cox et al., 2008).

**FIGURE 1:**
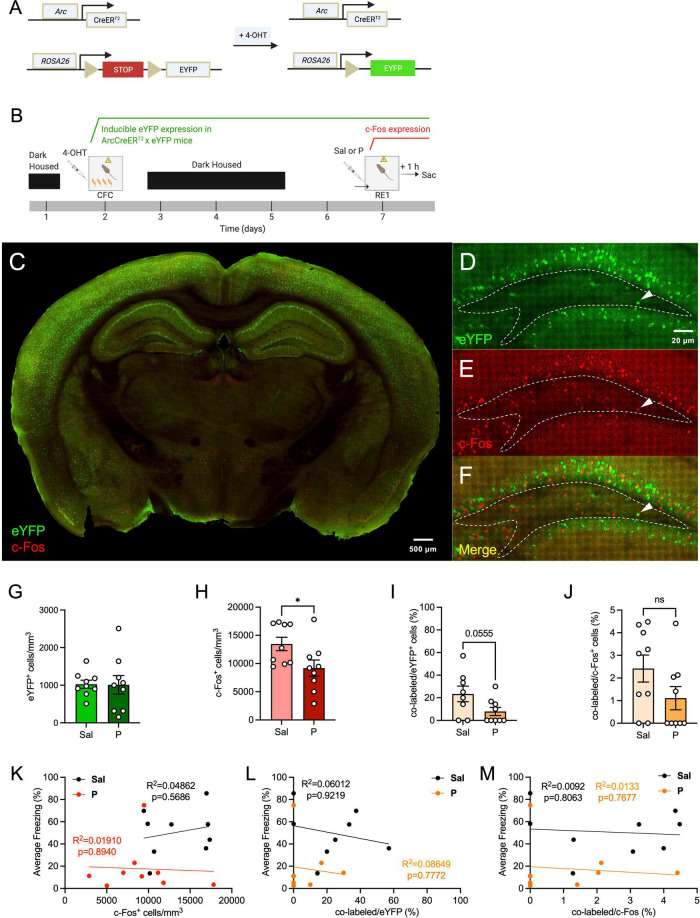
Propranolol administration decreases hilar neural activity in the dorsal hippocampus. **(A)** Genetic design. **(B)** Experimental design. 4-OHT was administered 5 h before CFC for neural tagging. Five days later, mice were given an injection of saline or propranolol. Immediately following the injection, mice were re-exposed the fear context and euthanized 1 h later. **(C)** Representative section showing eYFP (green) and c-Fos (red) expression throughout the brain in the ArcCreER^T2^ × eYFP mice. **(D)** eYFP^+^, **(E)** c-Fos^+^, and **(F)** co-labeled cells in the dorsal hilus. Representative hilar sections are outlined in white. **(G)** eYFP^+^, **(H)** c-Fos^+^, **(I)** co-labeled/eYFP (%), and **(J)** co-labeled/c-Fos^+^ (%) hilar cell counts. Correlation plots of average freezing vs. **(K)** c-Fos^+^ cells, **(L)** co-labeled/eYFP^+^ (%), and **(M)** co-labeled/c-Fos^+^ (%). Error bars indicate ± SEM. *p < 0.05, ^**^p < 0.01, ^***^p < 0.0001. eYFP, enhanced yellow fluorescent protein; 4-OHT, 4-hydroxytamoxifen; CFC, contextual fear conditioning; RE, context re-exposure; sac, sacrifice; Sal, saline; P, propranolol.

To determine how propranolol alters neural activity in the hilus, ArcCreER^T2^ x eYFP mice (Figure 1A) were injected with 4-OHT 5 h prior to 4-shock CFC (Figure 1B) to tag cells with eYFP. Five days later, mice were administered an injection of saline or propranolol prior to context re-exposure (RE) and euthanized 1 h following context RE to capture cells expressing c-Fos or Arc protein. As previously reported, propranolol administration significantly decreased fear expression during context RE when compared with saline administration (Leal Santos et al., 2021). The transgenic model allowed for brain-wide labeling of eYFP^+^ cells active during fear encoding (Figures 1C–F).

We first sought to determine whether administration of propranolol alters activity in the dorsal hilus. We found that expression of hilar eYFP^+^ cells did not differ between the saline and propranolol mice [*t*(16) = 0.06094, *p* = 0.9522] (Figure 1G), as expected since eYFP^+^ cells were tagged during CFC and before drug administration. Interestingly, the number of hilar c-Fos^+^ cells was significantly less in propranolol-injected mice when compared with saline-injected mice [*t*(16) = 2.311, *p* = 0.0345] (Figure 1H). Notably, this difference was not previously observed in the GCL of the dDG (Leal Santos et al., 2021).

When analyzing hilar memory trace reactivation, propranolol-injected mice administered had a trending, but not significant decrease in co-labeled/eYFP^+^ (%) of cells when compared with saline-injected mice [*t*(15) = 2.076, *p* = 0.0555] (Figure 1I). No difference was observed between the groups in the percentage of co-labeled/c-Fos^+^ cells [*t*(16) = 1.654, *p* = 0.1175] (Figure 1J). The lack of an effect in this metric may be due to the c-Fos^+^ cells being more variable than the eYFP^+^ population, encompassing not only excitatory cells but also inhibitory interneurons (Staiger et al., 2002; Tyssowski et al., 2018).

Next, we performed correlation analyses between the levels of hilar c-Fos expression and freezing levels, and between the percentage of hilar memory trace reactivation and freezing (Figures 1K–M). No correlation was found between the numbers of c-Fos^+^ cells and freezing levels for both groups (Figure 1K). When analyzing the correlation between hilar memory trace reactivation and freezing, we found no correlation between the percentage of co-labeled cells and freezing for both groups (Figures 1L,M). These data indicate that hilar memory trace activity does not directly reflect the behavioral levels of fear expression.

### Propranolol Administration Decreases Ventral Hilar Activity

**FIGURE 2:**
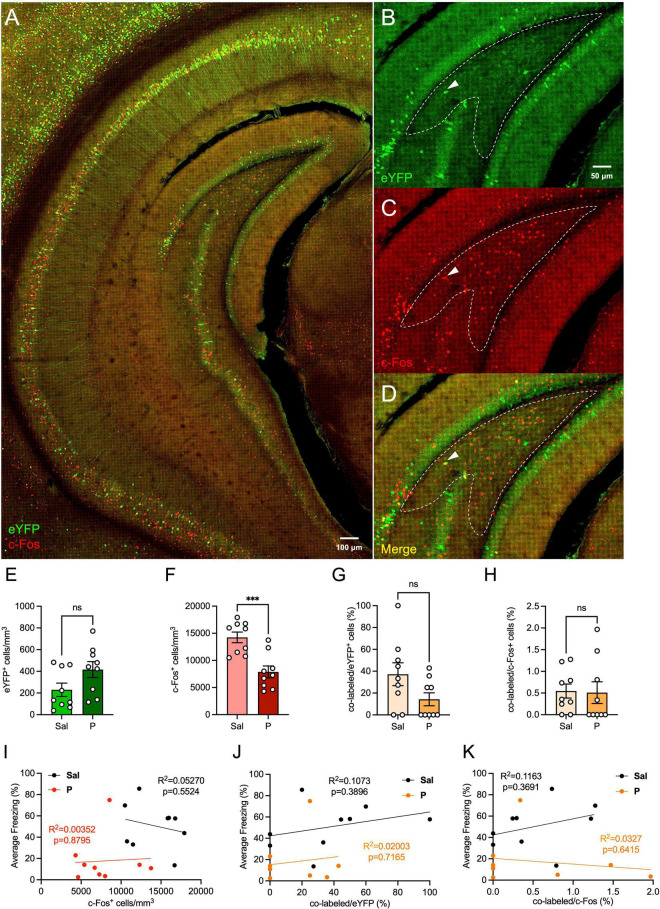
Propranolol administration decreases hilar neural activity in the ventral hippocampus. **(A)** Representative section showing eYFP (green) and c-Fos (red) expression throughout the brain in the ArcCreER^T2^ × eYFP mice. **(B)** eYFP^+^, **(C)** c-Fos^+^, and **(D)** co-labeled cells in the ventral hilus. Representative hilar sections are outlined in white. **(E)** eYFP^+^, **(F)** c-Fos^+^, **(G)** co-labeled/eYFP (%), and **(H)** co-labeled/c-Fos^+^ (%) hilar cell counts in the ventral hilus. Correlation plots of average freezing versus **(I)** c-Fos^+^ cells, **(J)** co-labeled/eYFP^+^ (%), and **(K)** co-labeled/c-Fos^+^ (%). Error bars indicate ± SEM. *p < 0.05, ^**^p < 0.01, ^***^p < 0.0001. eYFP, enhanced yellow fluorescent protein; Sal, saline; P, propranolol; ns, non significant.

We next sought to determine whether administration of propranolol alters activity in the ventral hilus (Figures 2A–D). We found that expression of hilar eYFP^+^ cells did not differ between the groups [*t*(16) = 1.965, *p* = 0.067] (Figure 2E). Interestingly, the number of hilar c-Fos^+^ cells was significantly lower in propranolol-injected mice when compared with saline-injected mice [*t*(16) = 4.336, *p* = 0.0005] (Figure 2F), as was seen in the dorsal hilus.

When analyzing hilar memory trace reactivation, propranolol-injected mice administered had comparable percentages of co-labeled/eYFP^+^ [*t*(16) = 1.911, *p* = 0.0742] (Figure 2G) and co-labeled/c-Fos^+^ [*t*(16) = 0.1253, *p* = 0.9019] (Figure 2H) when compared with saline-injected mice. Overall, these data suggest that ventral memory trace reactivation in the hilus is not altered following propranolol administration.

Finally, we performed correlation analyses between the levels of hilar c-Fos expression and freezing levels, and between the percentage of hilar memory trace reactivation and freezing (Figures 2I–K). No correlation was found between the numbers of c-Fos^+^ cells and freezing levels for mice administered either saline or propranolol (Figure 2I). When analyzing the correlation between hilar memory trace reactivation and freezing, we found no correlation between the percentage of co-labeled cells and freezing, for either the saline- or propranolol-injected mice (Figures 2J,K). These data indicate that hilar activity does not directly reflect the behavioral levels of fear expression, and this is not altered by propranolol administration.

### Propranolol Administration Does Not Alter Somatostatin Expression in the Hilus

We then hypothesized that propranolol could reduce dorsal hilar activity by enhancing the activity or expression of inhibitory interneurons. We next quantified the expression of somatostatin (SST) in the dorsal and ventral hilus (Supplementary Figures 1A,B). SST was chosen due to its demonstrated role in regulating long-term plasticity and hippocampal-dependent learning and memory, as well as its high co-expression with β-adrenergic receptors (Cox et al., 2008; Honoré et al., 2021). SST density was comparable between both groups in both the dorsal [*t*(16) = 1.223, *p* = 0.2389] (Supplementary Figure 1C) and ventral [*t*(16) = 0.0189, *p* = 0.9852] (Supplementary Figure 1E) hilus, indicating that propranolol administration does not significantly affect hilar SST expression.

We next performed correlation analyses between the levels of hilar SST expression and freezing levels (Supplementary Figures 1D,F). In the dorsal hilus, there was a positive correlation between freezing during RE and SST expression in propranolol-, but not saline-administered mice (Supplementary Figure 1C). Similarly, in the ventral hilus, there was a positive correlation between freezing during RE and SST in propranolol-, but not saline-injected mice (Supplementary Figure 1F). Together, these results suggest that although propranolol does not alter hilar SST expression, levels of SST^+^ interneurons may positively reflect the behavioral levels of fear expression in propranolol-administered mice.

### Propranolol Administration Increases the Activity of Parvalbumin-Expressing Inhibitory Cells in the Ventral, but Not Dorsal Hilus

**FIGURE 3:**
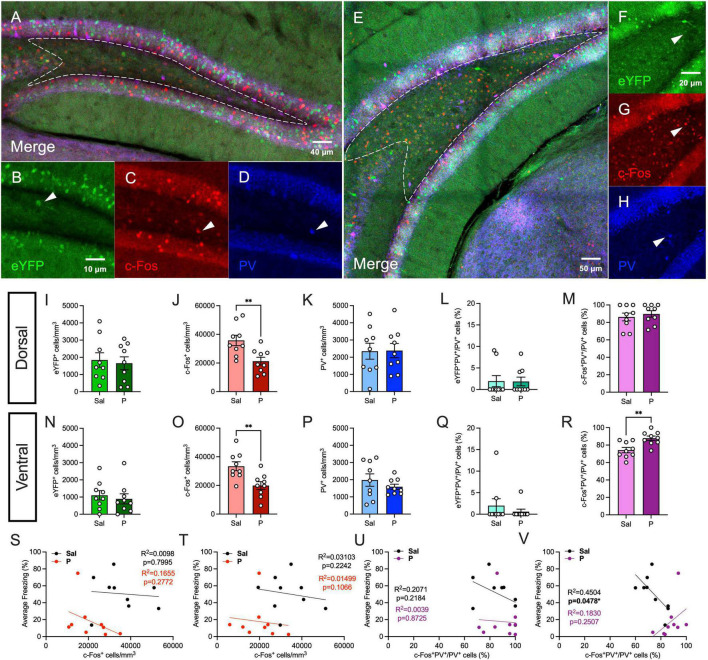
Propranolol administration increases the activity of parvalbumin-expressing cells in the ventral, but not dorsal hilus. **(A)** Representative section showing eYFP (green), c-Fos (red), and PV (blue) expression in the dorsal hippocampus. A representative hilar section is outlined in white. **(B)** eYFP^+^, **(C)** c-Fos^+^, and **(D)** PV^+^ cells in the dorsal hilus. **(E)** Representative section showing eYFP (green), c-Fos (red), and PV (blue) expression in the ventral hippocampus. A representative hilar section is outlined in white. **(F)** eYFP^+^, **(G)** c-Fos^+^, **(H)** PV^+^ cells in the ventral hilus. **(I)** eYFP^+^, **(J)** c-Fos^+^, **(K)** PV^+^, **(L)** eYFP^+^PV^+^/PV^+^ (%), and **(M)** c-Fos^+^PV^+^/PV^+^ (%) cells in the dorsal hilus. **(N)** eYFP^+^, **(O)** c-Fos^+^, **(P)** PV^+^, **(Q)** eYFP^+^PV^+^/PV^+^ (%), and **(R)** c-Fos^+^PV^+^/PV^+^ (%) cells in the ventral hilus. Correlation plots of average freezing vs. **(S)** c-Fos^+^ cells in the dorsal hilus, **(T)** c-Fos^+^ cells in the ventral hilus, **(U)** c-Fos^+^PV^+^/PV^+^ cells (%) in the dorsal hilus, and **(V)** c-Fos^+^PV^+^/PV^+^ cells (%) in the ventral hilus. Error bars indicate ± SEM. *p < 0.05, ^**^p < 0.01, ^***^p < 0.0001. eYFP, enhanced yellow fluorescent protein; PV, parvalbumin; Sal, saline; P, propranolol.

Next, we quantified hilar expression of parvalbumin (PV), another marker of inhibitory interneurons (Figures 3A–H). PV-expressing interneurons are GABAergic cells that mediate synchronous neural activity to promote memory consolidation, and their expression and activity can be significantly altered by stress or mood disorders (Ognjanovski et al., 2017; Chen et al., 2018; Perlman et al., 2021). PV is also co-expressed with β-adrenergic receptors in the hilus (Cox et al., 2008). As we previously demonstrated, in the dorsal hilus, propranolol significantly reduced c-Fos expression [*t*(16) = 3.179, *p* = 0.0058], but did not alter the expression of eYFP [*t*(16) = 0.3359, *p* = 0.7413] (Figures 3I,J). PV expression was comparable between both groups [*t*(16) = 0.0587, *p* = 0.9539] (Figure 3K). There was no significant difference in eYFP^+^PV^+^/PV^+^ (%) [*t*(16) = 0.597, *p* = 0.5589] or c-Fos^+^PV^+^/PV^+^ (%) [*t*(16) = 0.045, *p* = 0.9647] of cells between both groups (Figures 3L,M). These data suggest that propranolol reduces cellular activity in the dorsal hilus during re-exposure in a PV-independent manner.

In the ventral hilus, propranolol administration did not significantly alter eYFP expression [*t*(16) = 0.5123, *p* = 0.6154] (Figure 3N). As we previously demonstrated, propranolol significantly decreased c-Fos expression [*t*(16) = 0.3.247, *p* = 0.0051], but not PV expression [*t*(16) = 0.9995, *p* = 0.3324] (Figures 3O,P). eYFP^+^PV^+^/PV^+^ (%) [*t*(16) = 0.835, *p* = 0.416] of cells was comparable between saline- and propranolol-injected mice (Figure 3Q). Surprisingly, propranolol significantly increased the percentage of c-Fos^+^PV^+^/PV^+^ cells in the ventral hilus [*t*(16) = 3.418, *p* = 0.0035] (Figure 3R). Together, these data suggest that in the ventral hilus, propranolol increases the proportion of PV-expressing cells that are active during re-exposure. These findings are in line with previous data suggesting that increased inhibitory activity in vDG reduces stress-induced anxiety-like behaviors (Anacker et al., 2018).

Finally, we performed correlation analyses as described above (Figures 3S–V). c-Fos expression was not significantly correlated with freezing during RE in both the dorsal and ventral hilus, as previously demonstrated (Figures 3S,T). In the dorsal hilus, fear behavior was not correlated with c-Fos^+^PV^+^/PV^+^ (%) of cells (Figure 3U). In the ventral hilus, freezing was negatively correlated with the percentage of activated PV^+^ cells in saline-, but not propranolol-administered mice (Figure 3V). Together, these results suggest that the percentage of activated PV^+^ cells in the ventral hilus is negatively correlated with freezing behavior in controls, but that propranolol alters this behavioral correlation.

## Discussion

Here, we investigated how propranolol impacts activity in the hilus when administered prior to re-exposure to an aversive CFC context. We utilized the ArcCreER^T2^ x eYFP mice to allow tagging of the CFC encoding cells and to quantify reactivated cells following fear retrieval. We then examined the effect of acute administration of propranolol on SST and PV, two markers of inhibitory interneurons. We show that propranolol acutely: (1) decreases neural activity in the dorsal and ventral hilus, (2) alters the correlation between the activity of PV-expressing interneurons and freezing behavior, and (3) increases the activity of PV-expressing interneurons in the ventral, but not dorsal hilus.

We previously showed that propranolol decreased fear expression and decreased memory trace reactivation specifically in the dDG (Leal Santos et al., 2021). Studies of hippocampal connectivity have shown that information in the hippocampus flows mostly unidirectionally (Amaral et al., 2007; Bienkowski et al., 2018), with the DG being the entryway, and the hilus is the layer of the DG that receives the highest noradrenergic input (Pickel et al., 1974; Swanson and Hartman, 1975) and has discrete populations of cells expressing β-adrenergic receptors (Cox et al., 2008). These characteristics place the hilus as the layer most likely to be directly altered by propranolol, possibly relaying these effects to the GCL. Both β1- and β2-adrenoreceptors in the HPC have been extensively implicated in the modulation of memory formation (Qi et al., 2008). In rodents, β1- and β2-adrenoreceptors are expressed throughout the HPC, with higher levels of expression in the DG and lower levels of both receptor subtypes in CA3. These receptors are also expressed in interneurons and, to a lesser extent, in glial cells (Booze et al., 1993; Milner et al., 2000; Guo and Li, 2007; Cox et al., 2008). Here, we provide another layer of evidence of preferential noradrenergic modulation of the dDG upon retrieval of a contextual fear memory and show that β-adrenergic inhibition can decrease overall cellular activity in the hilus.

We explored the relation between either hilar activity and freezing during re-exposure, and found no correlation between these measures, suggesting that hilar activity alone cannot explain the behavioral effect of propranolol. In our paradigm, we previously reported a correlation between freezing and memory trace reactivation in the anterior cingulate area and in the lateral amygdala, but not in the GCL of the DG (Leal Santos et al., 2021). Memory trace reactivation in the DG has been shown to correlate with fear expression (Khalaf et al., 2018); however, the tagging systems and timelines differed between these studies, which might explain differences in engagement of the DG memory trace. Future analyses that encompass alterations across several regions and timepoints may yield a better understanding of network wide changes and of nodes that can more crucially impact the behavioral outcome (Vetere et al., 2017; Khalaf et al., 2018; Khalaf and Gräff, 2019; Leal Santos et al., 2021).

Here, we also combined our activity-based analysis with labeling for interneuron types (Houser, 2007; Cox et al., 2008), which can also be components of the memory trace (Trouche et al., 2013), in order to determine: (1) whether the change in overall activity was due to the excitatory or the inhibitory cell populations, or both, and (2) whether there is an inhibitory component of the memory trace in the hilus. Indeed, previous data indicate that modulation of noradrenergic inputs to the DG, resulting in altered contextual fear discrimination, can modulate the signaling of local interneuron populations (Seo et al., 2021). Here, we report that in propranolol-, but not saline-administered mice, there was a significant positive correlation between SST expression and freezing behavior, suggesting that the expression of SST interneurons could reflect behavioral output during fear memory retrieval. It has previously been demonstrated that SST is an important neuromodulator of hippocampal function at the behavioral level and that local hippocampal injections of SST can exert anxiolytic effects (Honoré et al., 2021). Additionally, we showed that propranolol administration can alter PV-expressing interneuron activity in the ventral, but not dorsal hilus. PV^+^ interneurons, particularly those localized in the hilar layer of the DG, exert fast-spiking inhibitory connections that are speculated to provide rapid suppression and to enable efficient higher-order network functions (Espinoza et al., 2018). Thus, by increasing the activity of inhibitory PV^+^ cellular input in the ventral hilus, propranolol may enable more efficient and powerful suppression of neuronal activity in the DG, thereby reducing the negative valence of the fear memory engram and suppressing freezing behavior. However, further study is necessary to fully characterize how propranolol alters the inhibitory component of a memory engram during memory retrieval.

Because PV^+^ interneurons express β-adrenergic receptors, it was unexpected that administration of propranolol, a β-adrenergic antagonist, would increase the percentage of cFos^+^PV^+^/PV^+^ cells during fear retrieval. It is known that within the hilus, different interneuron subtypes are interconnected and regulate each other’s activity (Sik et al., 1997). Therefore, whichever subpopulation of interneurons is more susceptible to propranolol may have a decrease in firing and subsequently disinhibit downstream interneurons, and this effect may predominate over the direct effect of the β-adrenergic antagonist to the latter interneurons. As quantified in Cox et al. (2008), PV^+^ interneurons are third to SOM^+^ neurons and then to NYP neurons in expression of β-adrenergic receptors. It is possible, therefore, that other populations of hilar interneurons undergo stronger inhibition by propranolol and disinhibit PV^+^ interneurons. Alternatively, although propranolol is a potent non-selective β-adrenergic antagonist, previous studies have shown that it may have partial agonist activity, leading to the activation of ERK pathways in β-adrenergic receptor-expressing cells (Azzi et al., 2003; Baker et al., 2003; Galandrin and Bouvier, 2006). Thus, although propranolol broadly acts to suppress the activity of β-adrenergic cells, in some cases, it may lead to cellular excitation due to activation of distinct GPCR signaling effectors. We speculate that this paradoxical upregulation of an intracellular signaling cascade may lead to increased activity of β-adrenergic receptor-expressing PV^+^ interneurons in the ventral hilus.

Another major cell type in the hilus is the mossy cell, which has unique structural and physiological properties. Mossy cells are glutamatergic neurons that have intrinsic and circuit properties that are thought to make them modulators of the activity of granule cells. Deletion of mossy cells leads to a transient increase in excitability and impaired contextual discrimination, and optogenetic activation of mossy cells primarily inhibits granule cells, suggesting that mossy cells primarily activate GABAergic interneurons that inhibit granule cells (as reviewed in Scharfman and Myers, 2012; Scharfman, 2016). c-Fos expression has been shown in mossy cells at baseline and under acute restraint stress (Duffy et al., 2013; Moretto et al., 2017). Although here we have initially focused on dissecting the activity of interneuron subtypes in the hilus, for a more complete understanding of how DG activity is affected by β-adrenergic modulation during fear retrieval, further studies will address how mossy cell activity is affected during β-adrenergic blockade and how the balance of inhibition and excitation between mossy cells and hilar interneurons modulate granule cell activity.

The hippocampal subfields display heterogeneity along their dorso-ventral axis in terms of gene expression, connectivity, and function (Moser and Moser, 1998; Bannerman et al., 2004; Fanselow and Dong, 2010; Kheirbek et al., 2013; Bienkowski et al., 2018). The dorsal HPC is necessary for learning and memory associated with spatial navigation (Moser et al., 1995; Bannerman et al., 1999; Leal Santos et al., 2021), whereas the ventral HPC is associated with innate fear and anxiety (Richmond et al., 1999; Kjelstrup et al., 2002). The dorsal HPC receives projections from sensory cortical areas and projects to associational cortical areas, while the vHPC is bidirectionally connected to regions such as the prefrontal cortex, amygdala and hypothalamus, implicated in the processing of emotional stimuli (Fanselow and Dong, 2010; Bienkowski et al., 2018). Interestingly, earlier we only reported an effect of propranolol in the dDG. However, here we report similar effects observed in the dorsal and ventral hilus for c-Fos, suggesting that propranolol may differentially impact hippocampal subregions and ultimately, fear behavior. However, in the ventral hilus, increased activity of a PV-expressing inhibitory population of interneurons could suppress the emotional valence of fear, perhaps by suppressing downstream excitatory projections to regions such as the basolateral amygdala (BLA) and prefrontal cortex (PFC). These combined effects could contribute to a significant overall reduction in freezing behavior in propranolol-administered mice at the time of memory retrieval.

In both human and animal studies that investigate how to improve propranolol’s efficacy and the neural mechanisms that mediate it (Fitzgerald et al., 2015; Giustino et al., 2016; Giustino and Maren, 2018), there has been difficulty establishing the boundary conditions in terms of timing, dosing, and stimulation that lead to a successful therapeutic outcome (Schroyens et al., 2017). Here, we show that, under certain conditions, propranolol’s effect on fear behavior is correlated with changes not only in the dDG, but now throughout the hilus. These data favor the use of propranolol for acute symptomatic relief. Where reconsolidation therapies are concerned, a greater understanding of the stimulation and dose parameters that suit this purpose is warranted. In this study, we have expanded the knowledge of the neurobiology underlying propranolol’s effect on fear behavior and identified the hilus as a target of noradrenergic modulation that may alter the recruitment of DG fear memory traces.

## Data Availability Statement

The raw data supporting the conclusions of this article will be made available by the authors, without undue reservation.

## Ethics Statement

All experiments were approved by the Institutional Animal Care and Use Committee (IACUC) at the New York Psychiatric Institute (NYSPI).

## Author Contributions

SL, BC, and CD contributed to the conception and design of the work, the analysis and interpretation of data for the work, drafting the work, and revising it critically for important intellectual content. SL, BC, GP, and VP contributed to the acquisition of data. SL, BC, GP, VP, and CD approved the version of the manuscript to be published and agreed to be accountable for all aspects of the work in ensuring that questions related to the accuracy and integrity of any part of the work are appropriately investigated and resolved. All authors contributed to the article and approved the submitted version.

## Conflict of Interest

SL, VP, and CD were employed by Research Foundation for Mental Hygiene, Inc. The remaining authors declare that the research was conducted in the absence of any commercial or financial relationships that could be construed as a potential conflict of interest.

## Publisher’s Note

All claims expressed in this article are solely those of the authors and do not necessarily represent those of their affiliated organizations, or those of the publisher, the editors and the reviewers. Any product that may be evaluated in this article, or claim that may be made by its manufacturer, is not guaranteed or endorsed by the publisher.
