# Supplementary material for: Propranolol Administration Modulates Neural Activity in the Hippocampal Hilus During Fear Retrieval
Source: Front Behav Neurosci. 2022 Jul 7;16:919831. doi: 10.3389/fnbeh.2022.919831 (PMC9301278; doi:10.3389/fnbeh.2022.919831)
Supplement: Supplementary file 1 [file Data_Sheet_1.pdf]

Supplementary Material

1 Supplementary Data

Not applicable.

2 Supplementary Figures and Tables

2.1 Supplementary Figures

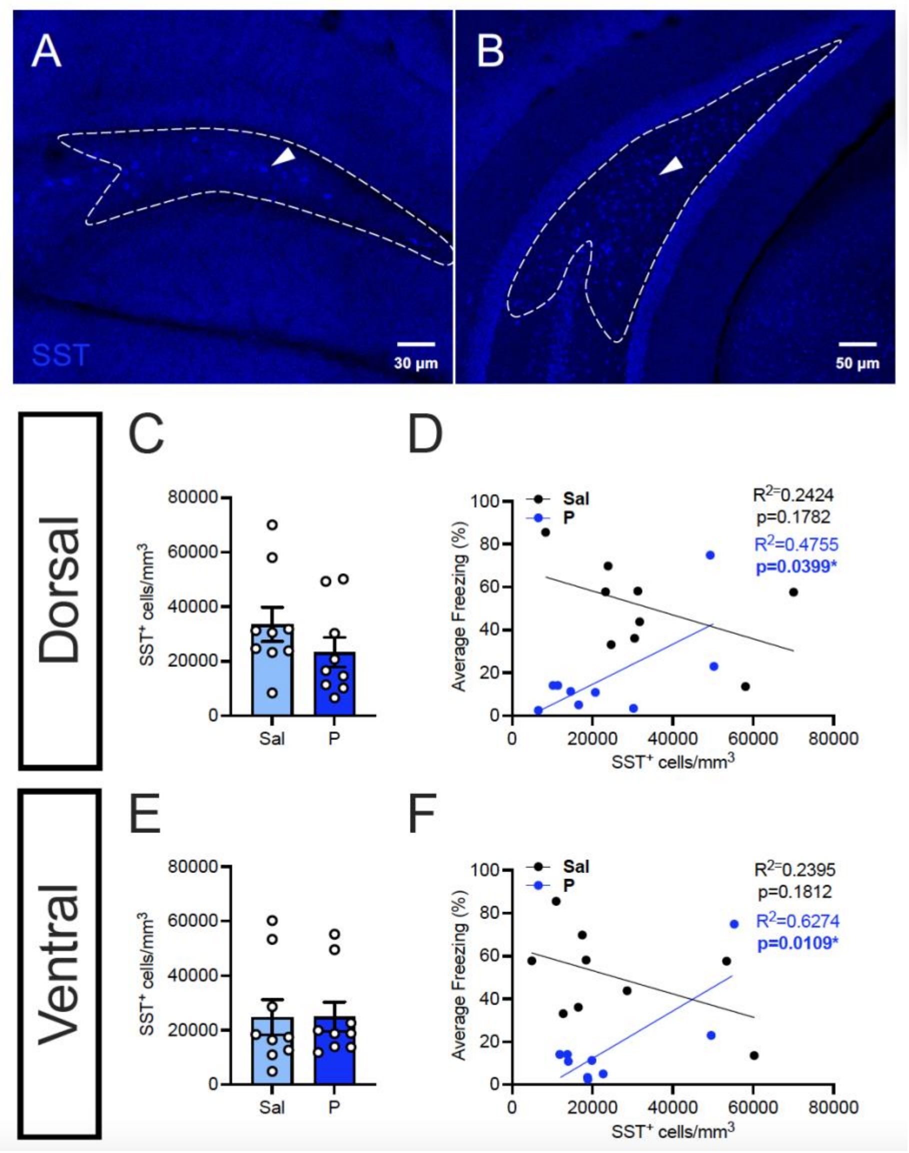

Supplementary Figure 1. Hilar somatostatin expression is significantly correlated with freezing during re-exposure in propranolol-injected mice. Representative section showing somatostatin (SST) (blue) expression in the (A) dorsal and (B) ventral hippocampus. Representative hilar sections

are outlined in white. (C) SST^+ cells in the dorsal hilus. (D) Correlation plots of average freezing versus dorsal hilar SST^+ cells. (E) SST^+ cells in the ventral hilus. (F) Correlation plots of average freezing versus ventral hilar SST^+ cells. SST, somatostatin; Sal, saline; P, propranolol.
