# Supplementary material for: Propranolol Administration Modulates Neural Activity in the Hippocampal Hilus During Fear Retrieval
Source: Front Behav Neurosci. 2022 Jul 7;16:919831. doi: 10.3389/fnbeh.2022.919831 (PMC9301278; doi:10.3389/fnbeh.2022.919831)
Supplement: Supplementary file 2 [file Table_1.pdf]

Table 1. Antibody Information

Primary Antibody	Primary Company	Primary Product No.	Primary Concentration	Primary RRID	Secondary Antibody	Secondary Company	Secondary Product No.	Secondary Concentration	Secondary RRID
Chicken anti-GFP	Abcam	13970	1:2000	AB_300798	Alexa Fluor® 488 ChromPure Chicken IgY (IgG)	Jackson Immuno	003-540-003	1:500	AB_2336976
Rabbit anti-c-fos	SySy	226003	1:5000	AB_2231974	Donkey anti-Rabbit IgG Alexa Fluor 555	Thermo Fisher Scientific	31572	1:500	AB_162543
Chicken anti-GFP	Abcam	13970	1:2000	AB_300798	Alexa Fluor® 488 ChromPure Chicken IgY (IgG)	Jackson Immuno	003-540-003	1:500	AB_2336976
Rabbit anti-c-fos	SySy	226003	1:5000	AB_2231974	Donkey anti-Rabbit IgG Alexa Fluor® 555	Thermo Fisher Scientific	31572	1:500	AB_162543
Rat anti-somatostatin	Millipore Sigma	MAB354	1:50	AB_2255365	Donkey anti-Rat IgG Alexa Fluor® 647	Abcam	150155	1:500	AB_2813835
Chicken anti-GFP	Abcam	13970	1:2000	AB_300798	Alexa Fluor® 488 ChromPure Chicken IgY (IgG)	Jackson Immuno	003-540-003	1:500	AB_2336976
Rat anti-c-fos	SySy	226017	1:5000	AB_2864765	Donkey anti-Rat IgG Alexa Fluor® 647	Abcam	150155	1:500	AB_2813835
Rabbit anti-parvalbumin	Swant	PV27	1:3000	AB_2631173	Donkey anti-Rabbit IgG Alexa Fluor® 555	Thermo Fisher Scientific	31572	1:500	AB_162543
