# Supplementary material for: Propranolol Administration Modulates Neural Activity in the Hippocampal Hilus During Fear Retrieval
Source: Front Behav Neurosci. 2022 Jul 7;16:919831. doi: 10.3389/fnbeh.2022.919831 (PMC9301278; doi:10.3389/fnbeh.2022.919831)
Supplement: Supplementary file 3 [file Table_2.pdf]

Table 2. Statistical Analysis

Cohort	Brain Region	Measurement	Comparison	t/R2	° of freedom	p	*	Figure
Arc/c-Fos	Dorsal Hilus	eYFP+ cells/mm3	Sal vs. P	0.06940	16	0.9522	ns	1G
		c-Fos+ cells/mm3	Sal vs. P	2.31100	16	0.0345	*	1H
		CL/eYFP+ cells (%)	Sal vs. P	2.07600	15	0.0555	ns	1I
		CL/c-Fos+ cells (%)	Sal vs. P	1.65400	16	0.1175	ns	1J
		Linear Regression: Average Freezing (%) vs. c-Fos+ cells/mm3	Sal	0.04862	1,7	0.5686	ns	1K
			P	0.01910	1,7	0.8940	ns	
		Linear Regression: Average Freezing (%) vs. CL/eYFP+ cells (%)	Sal	0.06012	1,6	0.5584	ns	1L
			P	0.08649	1,7	0.7772	ns	
		Linear Regression: Average Freezing (%) vs. CL/c-Fos+ cells (%)	Sal	0.009175	1,7	0.8063	ns	1M
			P	0.0133	1,7	0.7677	ns	
	Ventral Hilus	eYFP+ cells/mm3	Sal vs. P	1.96500	16	0.067	ns	2E
		c-Fos+ cells/mm3	Sal vs. P	4.33600	16	0.0005	***	2F
		CL/eYFP+ cells (%)	Sal vs. P	1.91100	16	0.0742	ns	2G
		CL/c-Fos+ cells (%)	Sal vs. P	0.12530	16	0.9019	ns	2H
		Linear Regression: Average Freezing (%) vs. c-Fos+ cells/mm3	Sal	0.05270	1,7	0.5524	ns	2I
			P	0.00352	1,7	0.8795	ns	
		Linear Regression: Average Freezing (%) vs. CL/eYFP+ cells (%)	Sal	0.10730	1,7	0.3896	ns	2J
			P	0.02003	1,7	0.7165	ns	
		Linear Regression: Average Freezing (%) vs. CL/c-Fos+ cells (%)	Sal	0.1163	1,7	0.3691	ns	2K
			P	0.03269	1,7	0.6415	ns	
Parvalbumin	Dorsal Hilus	eYFP+ cells/mm3	Sal vs. P	0.3359	16	0.7413	ns	3I
		c-Fos+ cells/mm3	Sal vs. P	3.179	16	0.0058	**	3J
		PV+ cells/mm3	Sal vs. P	0.05869	16	0.9539	ns	3K
		eYFP+PV+/PV+ cells (%)	Sal vs. P	0.04499	16	0.9647	ns	3L
		c-Fos+PV+/PV+ cells (%)	Sal vs. P	0.597	16	0.5589	ns	3M
	Ventral Hilus	eYFP+ cells/mm3	Sal vs. P	0.5123	16	0.6154	ns	3N
		c-Fos+ cells/mm3	Sal vs. P	3.247	16	0.0051	**	3O
		PV+ cells/mm3	Sal vs. P	0.9995	16	0.3324	ns	3P
		eYFP+PV+/PV+ cells (%)	Sal vs. P	0.835	16	0.4160	ns	EQ
		c-Fos+PV+/PV+ cells (%)	Sal vs. P	3.418	16	0.0035	**	3R
	Dorsal Hilus	Linear Regression: Average Freezing (%) vs. c-Fos+ cells/mm3	Sal	0.009844	1,7	0.7995	ns	3S
			P	0.1655	1,7	0.2772	ns	
	Ventral Hilus	Linear Regression: Average Freezing (%) vs. c-Fos+ cells/mm3	Sal	0.03103	1,7	0.2242	ns	3T
			P	0.01499	1,7	0.1066	ns	
	Dorsal Hilus	Linear Regression: Average Freezing (%) vs. c-Fos+PV+/PV+ cells (%)	Sal	0.2071	1,7	0.2184	ns	3U
			P	0.003942	1,7	0.8725	ns	
	Ventral Hilus	Linear Regression: Average Freezing (%) vs. c-Fos+PV+/PV+ cells (%)	Sal	0.4504	1,7	0.0478	*	3V
			P	0.183	1,7	0.2507	ns	
Somatostatin	Dorsal Hilus	SST+ cells/mm3	Sal vs. P	1.223	16	0.2389	ns	S1C
		Linear Regression: Average Freezing (%) vs. SSTs+ cells/mm3	Sal	0.2424	1,7	0.1782	ns	S1D
			P	0.4755	1,7	0.0399	*	
	Ventral Hilus	SST+ cells/mm3	Sal vs. P	0.01889	16	0.9852	ns	S1E
		Linear Regression: Average Freezing (%) vs. SST+ cells/mm3	Sal	0.2395	1,7	0.1812	ns	S1F
			P	0.6274	1,7	0.0109	*	
